# Mitochondrial IκBα fuels cancer progression through metabolic rewiring, endothelial activation, and thrombotic spread

**DOI:** 10.1038/s41420-026-03022-0

**Published:** 2026-03-27

**Authors:** Alessio Menga, Jessica Petiti, Roberta Basile, Pietro Poggio, Davide Acquarone, Alfonso Scalera, Lidia Avalle, Francesca Orso, Alessandra Bertoni, Paolo Ettore Porporato, Chiara Riganti, Lukasz Truszkowski, Isaia Barbieri, Mara Brancaccio, Carla Riera Domingo, Federica Cappellesso, Chiara Donno, Maiara Caroline Colombera, Massimiliano Mazzone, Giovanna Carrà, Alessandro Morotti

**Affiliations:** 1 Department of Health Sciences - Center for translational Research on Autoimmune & Allergic Diseases - CAAD, University of Eastern Piedmont, Novara, Italy; 2 Division of Advanced Materials Metrology and Life Sciences, Istituto Nazionale di Ricerca Metrologica (INRiM), Turin, Italy; 3 Department of Molecular Biotechnology and Health Sciences-Molecular Biotechnology Center “Guido Tarone”, University of Turin, Turin, Italy; 4 Department of Science and Technological Innovation (DISIT), University of Eastern Piedmont, Alessandria, Italy; 5 Department of Translational Medicine (DIMET), University of Eastern Piedmont, Novara, Italy; 6 Department of Oncology, University of Torino, Torino, Italy; 7 Molecular Biotechnology Center. Interdepartmental Center “G.Scansetti” for the study of asbestos and other toxic particulates, University of Torino, Torino, Italy; 8 Laboratory of Tumor Inflammation and Angiogenesis, Center for Cancer Biology, VIB, Leuven, Belgium; 9 Laboratory of Tumor Inflammation and Angiogenesis, Department of Oncology, Center for Cancer Biology, Leuven, Belgium; 10 Brussels center for Immunology, Vrije Universiteit Brussels, Brussels, Belgium; 11 Lab of Dendritic Cell Biology and Cancer Immunotherapy, VIB Center for Inflammation Research, Brussels, Belgium; 12 San Luigi Gonzaga Hospital, Regione Gonzole, Orbassano, Italy; 13 Department of Clinical and Biological Sciences, University of Turin, Turin, Italy

**Keywords:** Cancer microenvironment, Non-small-cell lung cancer, Cancer metabolism

## Abstract

Mitochondria play a central role in metastatic spread and cancer progression, with the IκBα/NF-κB signaling axis acting as a key regulator of both processes. We suggest that a stable fraction of IκBα localizes to mitochondria, where it escapes proteasomal degradation and acquires oncogenic functions independent of its canonical role in NF-κB inhibition. Using engineered A549 lung cancer cells with enforced mitochondrial localization of IκBα (IκBα-MTS), we show that the IκBα mitochondrial pool promotes increased cell proliferation, enhanced migration, and resistance to chemotherapy-induced apoptosis, along with a metabolic reprogramming characterized by elevated glycolysis and lactate secretion. These changes activated endothelial cells (ECs) and triggered cancer-associated thrombosis (CAT). This prothrombotic state, marked by elevated vWF a potent trigger for platelet adhesion and activation, contributed to an environment favorable for metastatic dissemination. Our findings reveal mitochondrial IκBα as a key mediator in mitochondrial stress, endothelial activation, and thrombo-inflammatory mechanisms that drive lung cancer progression.

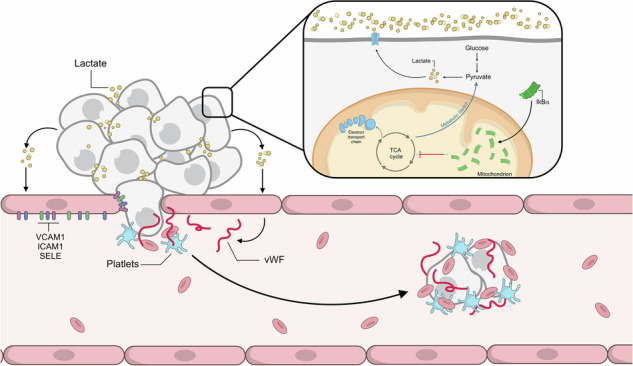

## Introduction

Metastasis remains the primary cause of cancer-related mortality and is no longer viewed as a process solely driven by intrinsic properties of cancer cells [1, 2]. Instead, successful metastatic colonization requires a coordinated remodeling of both the tumor and its microenvironment, including immune reprogramming, endothelial activation, and the establishment of a permissive vascular niche [3–5].

Central to this interplay is the metabolic plasticity of cancer cells, which not only sustains their growth but also actively reshapes the tumor microenvironment (TME). Metabolic rewiring, particularly the shift toward aerobic glycolysis (Warburg effect), leads to the production of lactate and other oncometabolites that contribute to immune evasion, angiogenesis, and matrix remodeling [6–8]. More recently, cancer metabolism has also been implicated in coagulopathy and thrombosis, phenomena collectively referred to as cancer-associated thrombosis (CAT) [9, 10]. The resulting prothrombotic state facilitates tumor cell adhesion, immune evasion, and dissemination, thus linking metabolism directly to metastatic potential [11].

Within this context, the NF-κB signaling axis and, in particular, its inhibitor IκBα, emerges as a multifaceted regulator of cancer cell fate. Classically known for its role in inflammation and immune responses, IκBα inhibits NF-κB by sequestering it in the cytoplasm under basal conditions, preventing its transcriptional activity. Interestingly, IκBα also exhibits unexpected mitochondrial localization and functions [12–17]. In several cancer models, including lung cancer, IκBα is enriched at the outer mitochondrial membrane (OMM), where it can modulate apoptotic signaling. Its mitochondrial accumulation is further influenced by hypoxic conditions, a hallmark of rapidly growing tumors. Despite increasing recognition of mitochondrial IκBα, its role in cancer cell metabolism and metastatic behavior remains poorly understood. We have recently shown that alterations in mitochondrial IκBα not only affect apoptotic thresholds but also drive metabolic dysfunction, leading to lactate accumulation and endothelial activation [18]. These changes are closely linked to the release of von Willebrand factor (vWF), a prothrombotic glycoprotein that contributes to endothelial permeability, inflammation, and ultimately cancer cell extravasation [19–25]. Emerging evidence suggests that thrombosis is not merely a complication of advanced malignancy but an active driver of metastasis formation [22, 26]. Tumor-induced endothelial activation and vWF release contribute to a self-reinforcing loop of vascular inflammation, coagulation, and metastatic seeding. In this study, we investigate the role of mitochondrial IκBα in orchestrating these events by integrating metabolic rewiring, prothrombotic signaling, and vascular remodeling. Our findings reveal a novel axis whereby mitochondrial IκBα enhances lung cancer cell aggressiveness through the promotion of a hypercoagulable, pro-metastatic microenvironment. This underscores a previously unrecognized link between mitochondrial signaling, cancer metabolism, and endothelial dysfunction in the pathogenesis of lung cancer metastasis.

## Results

### Mitochondrial targeting reveals a stable IκBα fraction

We previously reported that reduced IκBα expression in a cohort of lung cancer patients initially impairs tumor growth while inducing mitochondrial dysfunction [18]. Although our initial focus was on patients with low IκBα expression associated with enhanced chemosensitivity, a substantial subset of cases from the same cohort exhibited elevated IκBα levels [18].

**Fig. 1:**
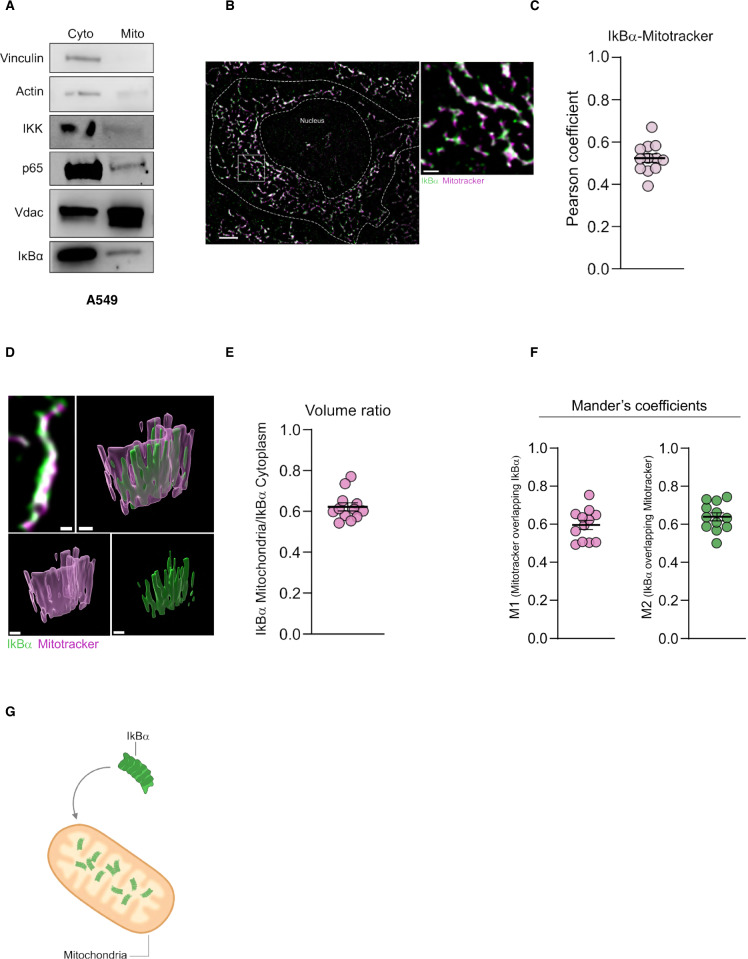
Mitochondrial targeting reveals a stable IκBα fraction. **A** Western blot analysis of cytoplasmic and mitochondrial subcellular fractionation of A549 cells, highlighting the presence of IκBα and its associated pathway within the mitochondria. **B** Images of A549 cells captured with the Elyra 7 Structured Illumination Microscope, along with a cropped zoomed-in view. In the images, IκBα is labeled in green, while the mitochondrial marker (mitotracker) is labeled in red. Scale bars: main image - 5 µm; zoom in - 1 µm. **C** Pearson correlation coefficient analysis was performed to assess the co-localization of IκBα with mitochondria. The results indicate a higher Pearson coefficient for IκBα in the mitochondrial compartment, suggesting localization or enrichment of IκBα within mitochondria in lung cancer cells. **D**, **E** Volume and volume ratio of IκBα levels in mitochondria compared to the cytoplasmic fraction. **F** Manders overlap coefficient (M1 and M2), indicating the fraction of IκBα signal overlapping with mitochondria and vice versa. Statistical significance of the observed colocalization was assessed by Costes randomization analysis. **G** Image depicting the mitochondrial localization of IκBα. All panels show endogenous IκBα unless otherwise indicated. Mitochondrial localization analyses were performed on cells expressing endogenous IκBα.

To better understand the role of IκBα overexpression, we performed subcellular fractionation of A549 lung cancer cells, and validated fraction purity using cytoplasmic and mitochondrial markers, confirming a strong enrichment. A549 cells were selected as they show elevated IκBα levels, allowing us to reveal the presence of IκBα and other pathway components within the mitochondria (Fig. 1A). Furthermore, data obtained with super-resolution microscopy confirmed IκBα localization inside mitochondria (Fig. 1B). The Pearson correlation coefficient indicates a colocalization between IκBα and the mitochondrial compartment (Fig. 1C). Quantitative analysis of IκBα volume and the volume ratio between the mitochondrial and cytoplasmic compartments further supports this observation (Fig. 1D, E). Finally, colocalization between IκBα and mitochondrial markers was quantitatively evaluated using the Manders overlap coefficient confirming a statistically significant association of IκBα with mitochondria, supporting its mitochondrial localization (Fig. 1F, G).

To explore the functional role of the mitochondrial IκBα/NF-κB axis, we engineered a fusion protein with a mitochondrial targeting signal (MTS) (residues 1–40 of the amino acid sequence of very long-chain acyl-CoA dehydrogenase) [27] at the N-terminus of IκBα. This construct was expressed generating a lentiviral vector, hereafter referred to as IκBα-MTS (Fig. S1a, Supporting Information).

The latter consistently exhibited exclusive IκBα mitochondrial expression, as evidenced by fluorescence microscopy imaging (Fig. S1A, Supporting Information). Confocal imaging revealed distinct localizations of our construct, demonstrating the presence of wild-type IκBα (IκBα-WT) in different cellular compartments, while IκBα-MTS was localized exclusively to the mitochondria (Fig. S1B, Supporting Information). Additionally, unlike IκBα-WT and endogenous IκBα, IκBα-MTS displayed significant stability following TNFα stimulation (Fig. S1C, Supporting Information). While both IκBα-WT and IκBα-MTS underwent phosphorylation, only the former was degraded (Fig. S1C, Supporting Information). This observation is consistent with the absence of canonical ubiquitin-proteasome activity within the mitochondrial compartment [28–30], which limits proteasomal degradation of mitochondrially targeted IκBα and thereby accounts for the increased stability of the MTS construct. Multiple published studies have underscored the presence of a phosphorylated form of IκBα [31] that remains stable, indicating the existence of an intracellular pool of IκBα resistant to degradation. This suggests its potential sequestration within organelles, such as mitochondria, where proteasome activity is diminished.

### Mitochondrial IκBα overexpression enhances the aggressiveness of lung cancer cells

**Fig. 2:**
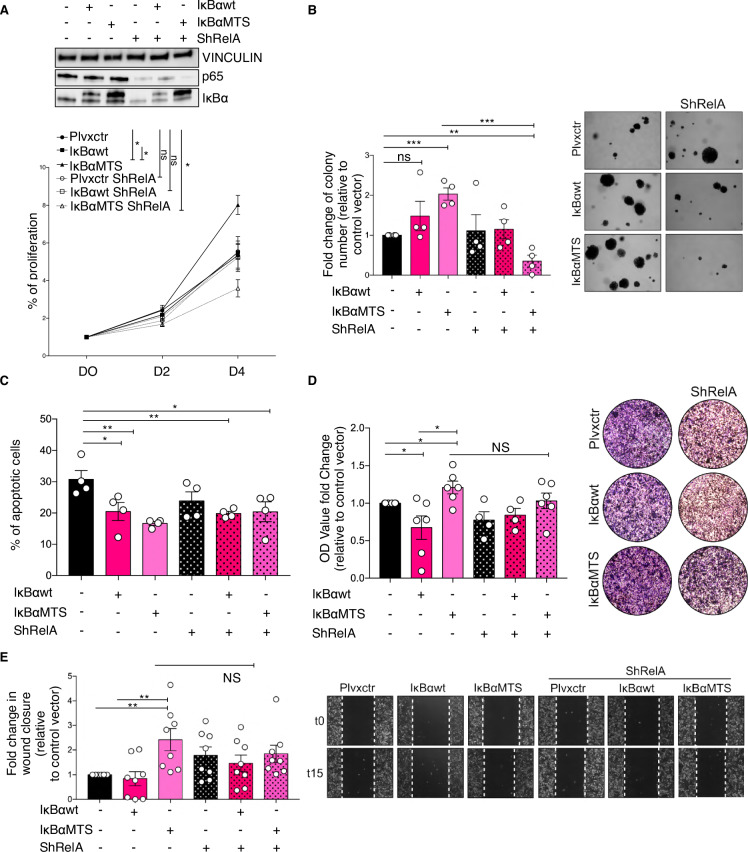
Mitochondrial IκBα overexpression enhances aggressiveness of lung cancer cells. **A** Upper panel: western blot analysis of IκBα and p65 in A549 cells demonstrating the overexpression of WT and MTS variants compared to control cells, along with p65 silencing. Lower Panel: Proliferation was evaluated over 4 days using the CellTiter Glo assay. Graph shows means ± SEM, *N* = 3 independent experiments, *P*-values are from Student’s t-test, with each condition compared to the control cells. *P < 0.05. **B** Colony formation assay, with representative images. Graph shows means ± SEM, *N* = 4 independent experiments. Statistical significance was determined using Student’s *t*-test, with WT and MTS conditions compared to control cells. **P < 0.01; ***P < 0.001; **C** Flow cytometry analysis of apoptotic induction following cisplatin treatment for 48 h. Graph shows means ± SEM, *N* = 4 independent experiments. Statistical significance was determined using Student’s *t*-test, with each experimental condition compared to cisplatin-treated control cells. *P < 0.05. **D** Invasion assay, shown as fold change relative to control cells. Representative images of cells that have invaded through the membrane, stained with crystal violet are shown. Graph shows means ± SEM, *N* = 6 independent experiments Statistical significance was determined using Student’s *t*-test, with WT and MTS conditions compared to control cells. *P < 0.05; **P < 0.01. **E** Left panel: fold change of wound closure, illustrating the progressive wound healing at 0 h and 15 h post wounding. Fold change relative to the control condition, normalized to 1. Right Panel: Representative pictures captured at the indicated time points to offer visual insight into the dynamic healing process. Graph shows means ± SEM, *N* = 8 independent experiments Statistical significance was determined using Student’s *t*-test, with WT and MTS conditions compared to control cells. **P < 0.01. WT and MTS indicate overexpression constructs for wild-type IκBα and mitochondrially targeted IκBα, respectively. “Control” refers to cells expressing endogenous IκBα only. Conditions including “shRELA” denote p65/RELA silencing used to assess NF-κB pathway contribution.

To assess the impact of mitochondrial IκBα on lung cancer aggressiveness, we overexpressed it in A549 and H460 lung cancer cell lines, with or without p65 silencing, in order to determine the role of NF-κB (Figs. 2A and S2A, Supporting Information). The overexpression of both IκBα -WT and -MTS resulted in increased cell growth (Fig. 2A, lower panel and Fig. S2B, Supporting Information) and colony formation (Fig. 2B). IκBα-WT cells displayed a similar trend, though less pronounced, suggesting that efficient mitochondrial accumulation, achieved *via* the MTS signal, maximizes this proliferative advantage. Notably, the silencing of p65 rescued both phenomena observed in IκBα-WT and IκBα-MTS constructs, indicating that canonical NF-κB components partially contribute to the proliferative phenotype. Consistent with our prior findings, the overexpression of IκBα-WT and -MTS also led to a reduced sensitivity to apoptosis induced by cisplatin treatment (Fig. 2C).

To investigate the impact of mitochondrial IκBα on the pro-metastatic behavior of lung cancer cells, we compared the phenotypic outcomes of IκBα-WT cells versus IκBα-MTS. Functional assays revealed that the expression of IκBα-MTS significantly enhanced cell motility and invasive capacity, hallmark traits of metastatic potential. In contrast, cells expressing IκBα-WT exhibited no notable changes in migration or invasion compared to controls (Figs. 2D, E and S2C, D, Supporting Information). These findings suggest that the subcellular localization of IκBα plays a critical role in modulating metastatic behavior, with mitochondrial IκBα acting as a specific driver of invasive phenotypes in lung cancer cells. In this context, p65 silencing appears insufficient to significantly rescue the phenotype.

### Mitochondrial IκBα favors metastatic spread and pro-coagulant state of TME

**Fig. 3:**
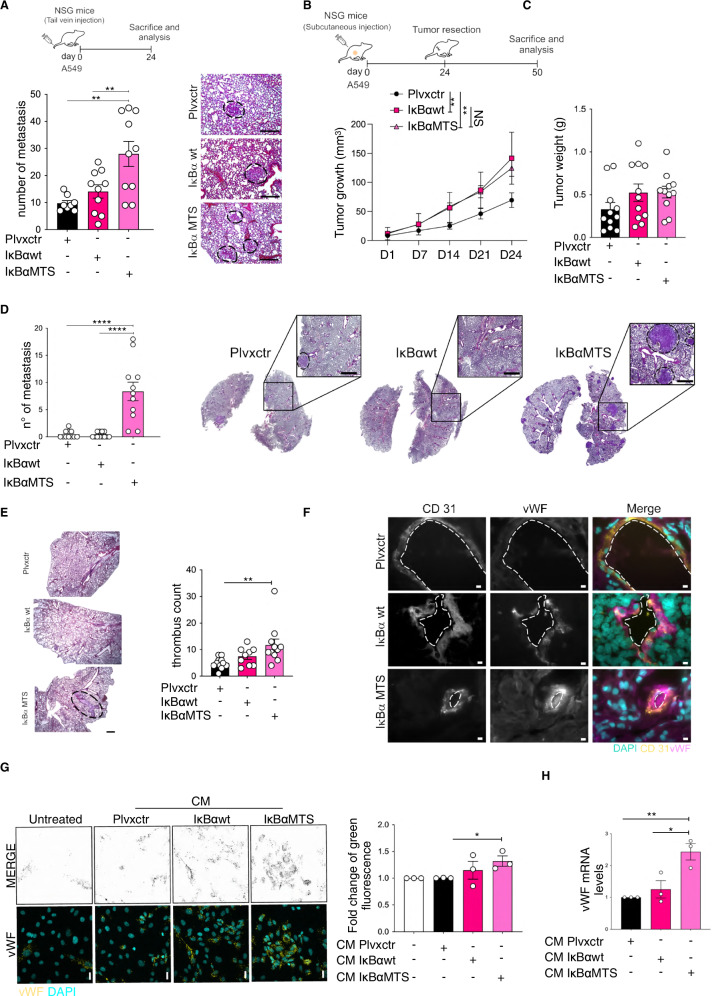
Mitochondrial IκBα favors metastatic spread and pro-coagulant state of TME. All in vivo experiments were performed using A549 cells infected with control, IκBα-WT, or IκBα-MTS overexpression vectors. **A** Upper panel: quantification of metastases obtained from the in vivo metastasis assay with A549 cells. Lower Panel: Representative images stained with hematoxylin and eosin (H&E) depict metastatic lesions, providing visual representation of metastatic spread in vivo. Data are presented as mean ± SEM from *N* = 10 mice per group. Statistical significance was determined by one-way ANOVA, with WT and IκBα-MTS groups compared to control mice. **P < 0.01. **B** Left panel: schematic representation illustrating the workflow of in vivo experiment. Nude mice were subcutaneously injected with IκBα -overexpressing A549 cells, after 24 days primary tumors were surgically dissected and mice sacrificed at day 50 to allow lung metastasis analysis. Right panel: tumor growth curve of mice injected with A549 cells infected with control vector, WT, or IκBα- MTS, measured by caliper every 7 days. Data are shown as mean ± SEM from *N* = 11 mice per group. Statistical significance was determined by one-way ANOVA, with WT and IκBα-MTS groups compared to control mice. **P < 0.01. **C** Tumor weight (grams) measured at the time of resection (day 24 post injection). Data are shown as mean ± SEM from *N* = 11 mice per group. **D** Left panel: quantification showing the number of metastases observed in the experimental mice 26 days after tumor resection. Statistical significance was determined by two-way ANOVA, assessing the effects of IκBα construct and experimental time point, between WT or IκBα-MTS and control groups. ****P < 0.0001. Right Panel: Representative images of whole lung sections stained with H&E, showing metastatic lesions. **E** Left panel: representative images stained with H&E illustrate microthrombi formation in lung vessels of mice injected subcutaneously with A549 cells carrying control vector, WT, or MTS IκBα. Right panel: Quantification of microthrombi. Data are shown as mean ± SEM from *N* = 11 mice per group. Statistical significance was determined by two-way ANOVA, with WT and IκBα-MTS groups compared to control mice. **P < 0.01. **F** Immunofluorescence staining was performed on tumor cryosections from A549 cells infected with control vector, WT, or MTS IκBα. DAPI was used to visualize cell nuclei (blue), CD31 for endothelial cells (red), and vWF (green). **G** Left panel: Immunofluorescence analysis of HUVECs treated with conditioned medium (CM) derived from A549 cells infected with control vector, WT, or MTS IκBα for 15 min. Right panel: Fold change quantification of vWF signal in HUVECs. Data are presented as mean ± SEM. Statistical significance was determined using Student’s t-test, with WT or IκBα-MTS CM compared to control CM. *P < 0.05. **H** Evaluation of vWF mRNA expression in HUVECs treated with indicated CM for 15 min. Graph shows means ± SEM, *N* = 3 independent experiments. Statistical significance was determined using Student’s *t*-test, with WT or IκBα-MTS CM compared to control CM. *P < 0.05; **P < 0.01.

Consistent with our in vitro findings, tail-vein injection of A549 cells into NSG mice revealed that while IκBα-WT cells showed no significant increase in lung metastasis, cells expressing mitochondrial IκBα-MTS exhibited significantly greater metastatic potential compared to both control and IκBα-WT cells (Fig. 3A).

The pro-metastatic effect of mitochondrial IκBα localization was further validated using a spontaneous metastasis assay. Nude mice were subjected to subcutaneous injections of IκBα-WT, IκBα-MTS, or control A549 cells. 24 days post-engraftment, tumors were surgically resected and analyzed, and mice were euthanized 30 days later (Fig. 3B, upper panel). Consistent with the in vitro data, both IκBα -WT and -MTS expression promoted an increase in primary tumor growth and size (Fig. 3B lower panel and 3C). However, concerning lung metastasis formation, an increase was observed exclusively in mice injected with IκBα-MTS-overexpressing lung cancer cells (Fig. 3D). These data, consistent with our previous observations (Fig. 2D, E), suggest a role for IκBα in promoting lung cancer aggressiveness. In particular, while IκBα-WT overexpression is insufficient to induce a pro-metastatic phenotype, mitochondrial IκBα may promote metastasis. Intriguingly, only mice injected with IκBα-MTS cells displayed increased blood clot formation in vessels around the metastatic sites (Fig. 3E). Tumors obtained from both IκBα-WT- and IκBα-MTS cells displayed more intense staining for the endothelial cells (ECs) marker CD31, along with atypical vessel structures compared to the A549 control-mice, but the distribution of the pro-hemostasis protein vWF within the vessels differed between the two IκBα-expressing cell lines (Fig. 3F). While in the IκBα-WT expressing tumors, vWF exhibited its typical staining pattern exclusively localized in the vessel wall, IκBα-MTS tumor tissue showed a notable increase in intravascular vWF abundance (Fig. 3F). Given the known effects of the thrombotic cascade on metastasis formation [32–34], this could explain the increased invasiveness of IκBα-MTS A549 cells compared to their IκBα-WT counterparts.

ECs synthesize and release vWF into the bloodstream [35]. To assess how mitochondrial IκBα-expressing lung cancer cells influence endothelial behavior, we exposed human umbilical vein endothelial cells (HUVECs) to tumor-conditioned medium (CM), simulating tumor–endothelium crosstalk in vitro. Interestingly, CM from IκBα-MTS expressing cells induced a rapid production of vWF by HUVECs, as indicated by immunofluorescence analysis (Fig. 3G), along with an increase in its mRNA expression (Fig. 3H).

### Mitochondrial IκBα triggers ECs activation

**Fig. 4:**
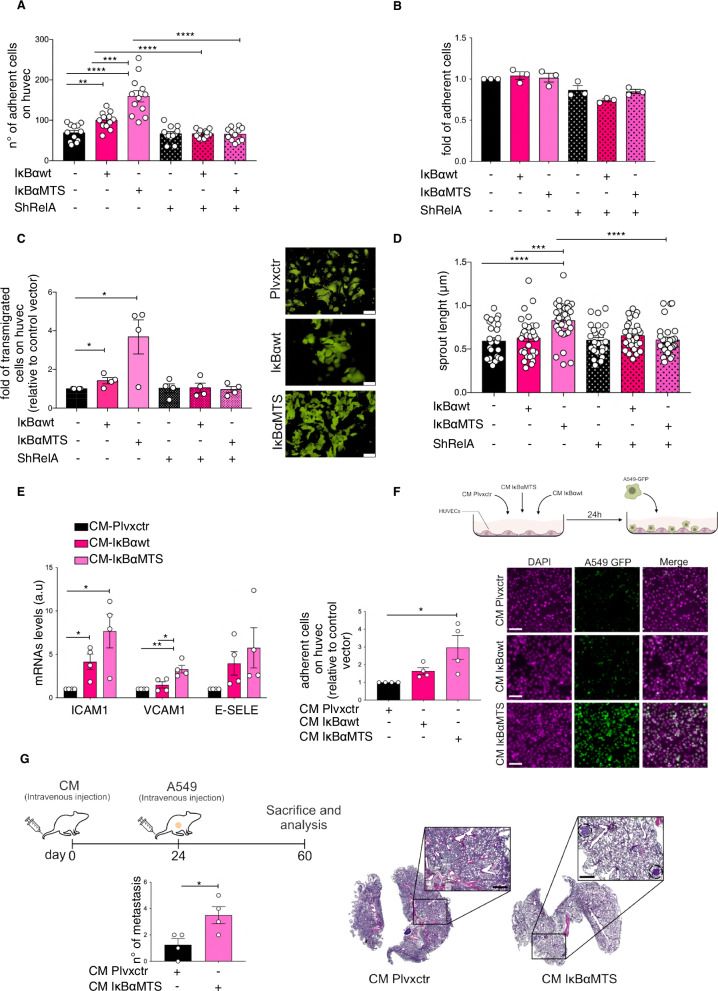
Mitochondrial IκBα triggers ECs activation. **A** Quantification of GFP-expressing A549 cells (Control, WT-IκBα, or MTS-IκBα, with or without p65 silencing) adherent on a HUVEC monolayer, as measured by FACS analysis. Graph shows means ± SEM, *N* = 3 independent experiments. Statistical significance was determined using Student’s t-test, with each experimental group compared to the corresponding control cells. **P < 0.01; ***P < 0.001; ****P < 0.0001. **B** Fold change in adhesion of lung cancer cells relative to control, as described in A, on plates. Graph shows means ± SEM, *N* = 3 independent experiments. Statistical significance was determined using Student’s *t*-test, with each condition compared to control cells. **C** Left panel: Fold change of lung cancer cells described in A relative to control, illustrating their migratory behavior. Graph shows means ± SEM, *N* = 4 independent experiments. Statistical significance was determined using Student’s t-test, with each condition compared to control cells. *P < 0.05. Right panel: Representative images showing GFP+ lung cancer cells transmigrated through a HUVEC monolayer. **D** Measurement of sprout length induced by conditioned medium (CM) derived from the cell lines described in A. Graph shows means ± SEM, *N* = 3 independent experiments statistical significance was determined using Student’s t-test, with each CM treatment compared to control CM. ***P < 0.001; ****P < 0.0001. **E** Quantitative RT-PCR analysis performed on HUVECs treated with CM from A549 cells for 24 h, which were infected with control vector, WT, or MTS IκBα. Data are mean ± SEM from *N* = 3 independent experiments. Statistical significance was determined using Student’s t-test, with each CM condition compared to control CM. *P < 0.05; **P < 0.01. **F** Upper panel: Schematic representation of experimental setup. HUVECs were pre-treated with CM from IκBα overexpressing A549 for 24 h, then GFP-positive A549 were plated on a HUVEC monolayer to assess their adhesion potential. Lower left panel: Fold change in adherent GFP-positive A549 cells adherent on HUVEC cells after treatment with the indicated CM for 24 h. Data are mean ± SEM from *N* = 4 independent experiments. Statistical significance was determined using Student’s t-test, with each CM treatment compared to control CM. *P < 0.05. Lower Right panel: Representative images of adherent GFP-positive A549 cells on the HUVECs cell layer. **G** Upper panel: schematic representation of experimental setup. Lower Left panel: Quantification of lung metastases in mice preconditioned with the indicated CM prior to injection with parental A549 cells. Data are mean ± SEM from *N* = 4 mice per group. Statistical significance was determined using one-way ANOVA, with WT or MTS CM-preconditioned mice compared to control CM. *P < 0.05. Lower Right panel: Representative pictures of lungs stained with H&E highlighting metastatic lesions. “Control” indicates cells expressing endogenous IκBα only. WT and MTS indicate overexpression constructs. Where indicated, “shRELA” denotes RELA/p65 silencing. Conditioned medium (CM) was collected separately from each condition and applied to HUVECs as specified.

ECs represent the initial barrier that prevents cancer cells from entering the bloodstream and reaching distant sites [36]. Hence, we investigated the functional interconnection between lung cancer cells and ECs. IκBα-MTS cells adhered significantly more to an HUVEC monolayer compared to the control and IκBα-WT lung cancer cell lines (Fig. 4A), while no differences in adhesion were detected in the absence of ECs (Fig. 4B). On the other hand, transmigration experiments revealed the capability of lung cancer cells overexpressing IκBα-MTS to migrate across ECs (Fig. 4C). Moreover, CM derived from IκBα-MTS cells significantly enhanced vessel sprouting and tube formation, suggesting a critical role in angiogenesis and vascular network development (Figs. 4D and S3A, B, Supporting Information). These changes were significantly less prominent with CM from IκBα-WT cells, indicating that these functions are predominantly linked to the mitochondrial localization of IκBα, as previously shown.

To further investigate the crosstalk between lung cancer cells and ECs, we assessed the ability of IκBα engineered lung cancer cells to induce the activation of HUVECs by triggering the expression of adhesion proteins on the EC surface. Upon treating HUVECs with CM from IκBα-expressing cells, qRT-PCR analysis revealed increased expression of RNAs encoding adhesion markers, such as VCAM1, ICAM1, and SELE (Fig. 4E). In line with our previous observation, we showed a significant increase in adhesion of parental A549 cells to HUVECs following treatment with IκBα-MTS CM (Fig. 4F, left and right panels).

To confirm these data in vivo, we preconditioned mice by intravenously injecting them with CM from cells expressing IκBα every two days for 24 days (Fig. 4G, upper panel), followed by intravenous injection of parental A549 cells. Notably, mice receiving CM derived from IκBα-MTS cancer cells developed significantly more metastases compared to mice treated with IκBα-WT or parental A549 CM (Fig. 4G, lower panel). These data suggest that lung cancer cells overexpressing IκBα in the mitochondria can alter the premetastatic niche (PMN) through the secretion of extracellular factors.

### Mitochondrial IκBα induces metabolic reprogramming in lung cancer cells

**Fig. 5:**
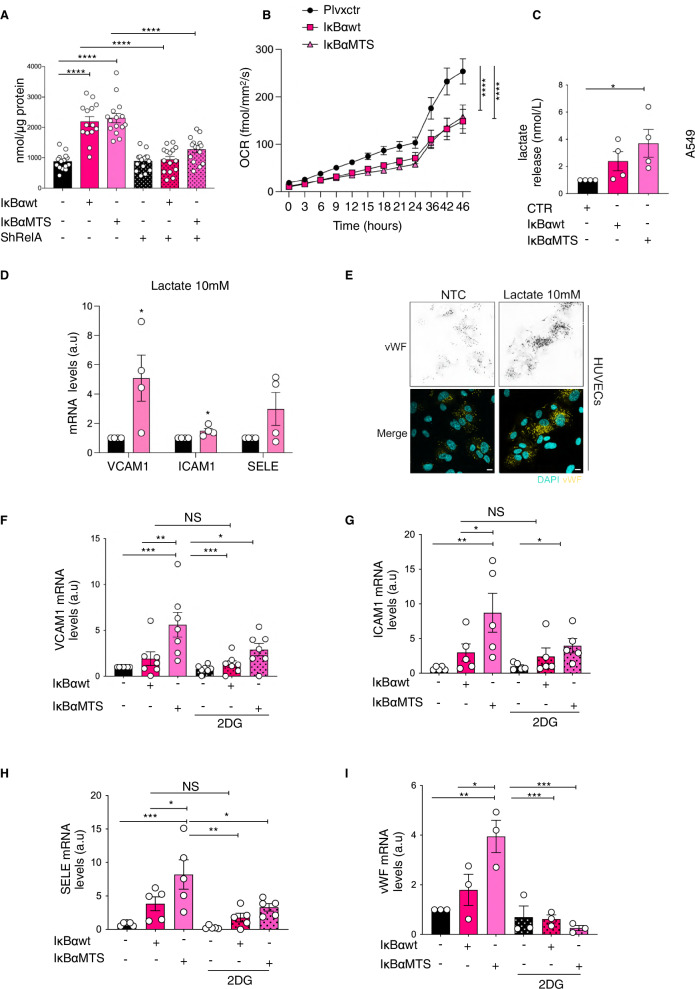
Mitochondrial IκBα induces metabolic reprogramming in lung cancer cells. Metabolic assays were performed on A549 cells expressing endogenous IκBα (Control), or overexpressing IκBα-WT or IκBα-MTS. In panels where indicated, p65/RELA silencing (shRELA) was used to assess NF-κB dependency. **A** Quantification of glucose uptake in A549 cells infected with control vector, WT, or MTS IκBα and silenced or not for p65. Cells were incubated with [³H]-glucose for 2 h. After incubation, medium was collected and radioactivity was measured using liquid scintillation counting, indicating the rate of glucose uptake and utilization by the cells. Data are mean ± SEM from *N* = 3 independent experiments. Statistical significance was determined by one-way ANOVA, with WT or MTS ± shRELA compared to corresponding control cells. ****P < 0.0001. **B** Oxygen flux of A549 cells described in A measured using the RESIPHER. Data were sampled every 3 h to monitor oxygen consumption rates. Data are mean ± SEM from *N* = 3 independent experiments. Statistical significance was determined by two-way ANOVA, with WT or MTS compared to control cells. ****P < 0.0001. **C** Lactate concentrations in the culture media were measured after 48 h of incubation of A549 cells infected with control vector, WT, or MTS IκBα. Cells were cultured under normoxic conditions. Lactate release was quantified using Biosen C. Values are mean ± SEM from *N* = 3 independent experiments. Statistical significance was determined by one-way ANOVA, with WT or MTS compared to control cells. *P < 0.05. **D** Quantitative RT-PCR analysis performed on HUVECs treated with different concentration of lactate for 15 min. Data are mean ± SEM from *N* = 4 independent experiments. Statistical significance was determined by one-way ANOVA compared to untreated HUVECs. ****P < 0.0001. **E** Immunofluorescence analysis of HUVECs treated with 10 mM lactate for 15 min. **F**–**I** Quantitative RT-PCR analysis was conducted on HUVECs exposed to conditioned medium (CM) derived from A549 cells for 24 h. The A549 cells were infected with control vector, WT, or MTS IκBα, and subsequently treated or untreated with 8 mM 2-deoxyglucose (2DG) for 4 h prior to collecting the CM. Data are mean ± SEM from *N* = 8 independent experiments. Statistical significance was determined by one-way ANOVA, with each CM condition compared to control CM. *P < 0.05; **P < 0.01; ***P < 0.001.

The behavior of neighboring cells is affected by changes in the tumor microenvironment, which in turn is shaped by alterations in cancer cell metabolism [37, 38]. To assess potential metabolic changes in engineered lung cancer cells that could impact ECs, we conducted experiments to measure the rate of glucose uptake and catabolism by exploiting the radioactive [H3] glucose assay. After a 2-h incubation period, the medium was collected, and the radioactivity was measured. As shown in Fig. 5A, engineered cells exhibited an increase in glucose uptake and catabolism. In agreement with this observation, IκBα-overexpressing A549 cells, when subjected to measurements of oxygen consumption overtime, showed reduced respiration (Fig. 5B); however, only cells expressing the mitochondrial IκBα exhibited a significant increase in lactate release into the medium (Fig. 5C).

Importantly, the TME is often characterized by acidic conditions due to increased lactate production resulting from enhanced glycolysis [8]. Lactate serves as a signaling molecule, stabilizing pathways such as HIF (Hypoxia-Inducible Factor), which regulate angiogenesis-related gene expression. It also stimulates EC functions crucial for angiogenesis, including proliferation, migration, and tube formation [8]. Recent studies have highlighted the role of lactate in modulating EC behavior, including the release of vWF [39, 40]. Consistent with these findings, treatment of HUVECs with 10 mM lactate for 15 min resulted in an increase in endothelial adhesion markers and vWF, as shown in Fig. 5D, E.

To illustrate the involvement of glycolytic metabolism in EC activation, we conducted an experiment using acute exposure (4 h) to 2-deoxy-D-glucose (2DG), a glucose analog that selectively targets glucose metabolism while limiting secondary cellular stress. Pretreating engineered A549 cells with 2DG reduced the levels of pro-adhesive and pro-thrombotic molecules in ECs exposed to their conditioned medium. As shown in Fig. 5F–I, CM derived from IκBα-MTS lung cancer cells significantly reduced the expression of adhesion markers (Fig. 5F–H) and vWF accumulation (Fig. 5I). This metabolic change in lung cancer cells, induced by IκBα-MTS overexpression, could explain EC activation and potentially the ability of cancer cells to facilitate the formation of the metastatic niche [41–43].

## Discussion

Our study sheds new light on the intricate connection between mitochondrial IκBα, lung cancer cell metabolism, and its profound impact on the TME, particularly affecting ECs and CAT [44, 45]. Previous research has established the multifaceted role of IκBα/NF-κB in modulating redox homeostasis in lung cancer cells. Expanding upon this knowledge, our findings revealed distinct phenotypes in lung cancer cells depending on varying expression levels and subcellular localization of the IκBα protein [18].

Unlike in the cytoplasm, where IκBα undergoes rapid degradation following Ser32-Ser36 phosphorylation, mitochondrial IκBα appears resistant to this degradation, allowing for its accumulation. This suggests that overexpressed IκBα, when accumulated in mitochondria, may acquire novel biological functions separate from its canonical role in cytoplasmic NF-κB inhibition. Indeed, mitochondrial IκBα functions appear entirely distinct from its well-known cytoplasmic role in controlling p65/NF-κB activity. Instead, within the mitochondria, IκBα seems to adopt an oncogenic role, significantly boosting the metastatic potential of lung cancer cells. This enhanced metastatic capacity could stem from an increase in circulating tumor cells or more efficient early lung metastasis formation. This interpretation aligns with our in vitro RELA-silencing experiments, in which proliferative defects are rescued whereas metastatic behavior is not fully reversed, suggesting that mitochondrial IκBα mediates biological functions beyond canonical NF-κB regulation, potentially through alternative molecular partners.

Furthermore, the tumor resection process may have initiated a wound healing response [46], promoting a pro-inflammatory environment favorable to the proliferation and dissemination of cancer cells, particularly those with heightened metastatic potential, such as IκBα-MTS cells. This consideration acquires further relevance within the framework of an inflammatory response, which may be linked to coagulation phenomena triggered by the release of vWF and the formation of microthrombi. These features were notably accentuated in IκBα-MTS primary tumors and metastases. This observation suggests the need for a closer examination of the development of CAT, which poses significant risks for cancer patients [47]. The intricate interplay between CAT and metastasis encompasses various interconnected mechanisms: a hypercoagulable state due to the release of procoagulant factors [48]; platelet activation facilitating tumor cell adhesion and protection [49, 50]; enhanced angiogenesis; interaction with the coagulation system promoting tumor growth; and inflammatory responses contributing to thrombosis through cytokine-mediated pathways [51]. Moreover, endothelial dysfunction further exacerbates thrombus formation, facilitating cancer cell extravasation and metastasis formation [52].

Our data suggest that mitochondrial IκBα contributes to metabolic reprogramming, with aerobic glycolysis, and particularly lactate, being a central player in aggressiveness. Indeed, IκBα-MTS cells showed significantly increased extracellular lactate accumulation. This finding suggests alterations in lactate handling rather than glycolytic engagement per se, potentially involving differences in lactate export through monocarboxylate transporters (e.g., MCT1/MCT4), changes in pyruvate flux, or mitochondrial activity influencing the balance between lactate production and oxidative metabolism. Addressing these possibilities will require targeted approaches, including MCT inhibition, direct comparisons between lactic acid and sodium lactate, and controlled buffering strategies, which will be essential to disentangle lactate transport-dependent versus pH-driven mechanisms and to further define the metabolic role of mitochondrial IκBα [53].

Notably, lactate release is not only linked to endothelial activation and angiogenesis but also plays a key role in inducing platelet aggregation and enhancing pro-thrombotic activity [54]. While our findings provide strong evidence for lactate as a key signaling molecule driving endothelial activation and the prothrombotic state, we do not exclude the possibility that other secreted metabolites, resulting from the observed mitochondrial dysfunction, could act in concert to shape the pro-metastatic niche. Further metabolomic studies could clarify the full spectrum of factors involved in this crosstalk [55].

A key question arising from our study is the direct molecular mechanism by which mitochondrial IκBα impairs oxidative phosphorylation. Identifying its specific binding partners within the mitochondrial proteome is therefore a critical future endeavor to fully elucidate how it triggers metabolic rewiring. One possibility is that IκBα interacts with components of the electron transport chain, mitochondrial metabolic enzymes, or other resident mitochondrial proteins, thereby altering their activity or assembly rather than their expression. Alternatively, mitochondrial IκBα may modulate mitochondrial signaling pathways or protein-protein interactions that indirectly affect respiratory efficiency. These possibilities remain to be experimentally addressed.

It is well established that primary tumors can prime the PMN to facilitate the colonization of disseminated cancer cells, yet the mechanisms by which dormant vasculature is activated at distant sites remain poorly defined. In this context, we investigated the ability of IκBα-MTS-expressing lung cancer cells to modulate ECs and potentially initiate vascular activation in secondary sites, thereby contributing to metastasis formation.

Our findings indicate that mitochondrial IκBα in lung cancer cells disrupts EC homeostasis. Through paracrine signaling, mitochondrial IκBα primary tumors exert influence over distant organs, promoting vascular remodeling and preparing a permissive PMN for circulating cancer cells. These processes collectively enhance lung cancer cell intravasation and metastasis.

Nevertheless, several aspects of this pathway remain to be elucidated. For instance, the mechanism by which IκBα enters the mitochondria warrants further investigation. It is possible that its mitochondrial translocation is mediated by hypoxia and STAT3 signaling pathways [56]. Additionally, recent findings have identified irregularities in MTS sequences [57], suggesting the potential for alternative splicing or mutations affecting IκBα localization.

Importantly, under What is clear, however, is that, under certain specific conditions, particularly e.g. in oncogenic [58] or hypoxic environments [56], IκBα can localize to the mitochondria. Our data suggest a dual role for mitochondrial IκBα. While its effects on cell proliferation and colony formation appear to remain dependent on the p65/NF-κB axis, as indicated by our p65 silencing experiments, the profound metabolic shift towards glycolysis and the subsequent activation of the endothelium appear to be direct consequences of its mitochondrial localization, representing a novel, non-canonical function. Thus, mitochondrial IκBα may contribute to cancer progression through both NF-κB-dependent and independent mechanisms, potentially conferring gain-of-function properties that promote tumor growth and metastasis [59].

Altogether, our findings suggest that mitochondrial localization of IκBα may significantly contribute to the metastatic burden in lung cancer. While RELA silencing provided important insight into pathway dependency, more refined NF-κB functional assays will be necessary to fully delineate canonical versus non-canonical contributions. Importantly, we did not directly quantify NF-κB transcriptional activity in this study; future reporter-based or transcriptomic approaches will be necessary to more precisely define the canonical versus non-canonical contributions to the phenotypes associated with mitochondrial IκBα. Furthermore, as IκBα is a canonical inhibitor of NF-κB, the mitochondrially targeted form used here does not directly engage the cytoplasmic NF-κB regulatory cycle, and therefore its biological effects cannot be interpreted as simple NF-κB inhibition. These limitations outline important avenues for future investigation to disentangle mitochondrial and cytoplasmatic IκBα. Finally, an important limitation of the study is the reliance on overexpression systems to manipulate mitochondrial IκBα localization. Tools to selectively regulate mitochondrial import of endogenous IκBα are not currently available, and future models will be required to confirm these findings under physiological conditions.

Nonetheless, our findings collectively reveal a potential novel function of IκBα that operates independently of canonical NF-κB signaling. This non-canonical, mitochondria-associated activity may bypass compensatory inhibitory pathways and highlights new therapeutic opportunities aimed at the mitochondrial pool of IκBα or its downstream metabolic and thrombotic effectors.

## Material and methods

### Cell lines

The human lung cancer cell lines A549 (RRID: CVCL_0023) and H460 (RRID: CVCL_0459) were purchased from ATCC. The cells were cultured in RPMI 1640 medium supplemented with 10% FBS, 1% L-glutamine, and 1% penicillin/streptomycin. All cell lines were maintained in a humidified incubator with 5% CO_2_ at 37 °C. HUVECs, sourced from pooled donors, were acquired from PromoCell (Cat.No.C-12205) and cultivated in dishes coated with 0.2% gelatin, utilizing Endothelial Cell Growth Medium 2 (EGM2) enriched with provided growth factors (PromoCell, C-22011), penicillin (100 U/ml), and streptomycin (100 μg/ml; Gibco). HUVECs were utilized between passages 3 and 7, with medium renewal occurring every 48 or 72 h. The cells were routinely tested and confirmed to be free of mycoplasma and bacterial contamination.

### Cell proliferation assay and apoptosis assessment

For the cell proliferation assay, cells were plated in 96-well plates at a density of 2 × 10^3^ cells per well. Proliferation was measured using the CellTiter-Glo assay (Promega). Apoptosis was assessed by flow cytometry after Annexin V staining (Biolegend, #640920). Data acquisition and analysis were performed using a FACSCelesta cytometer and CellQuest software. Both assays were performed according to the manufacturers’ instructions.

### MitoTracker

For MitoTracker staining (Invitrogen, #M7510), cells were seeded at a confluence of 2.5 × 10^5^ cells per well in 6-well plates. MitoTracker was then added to the culture medium at a final concentration of 10 nM and incubated for 15 minu at 37 °C. Subsequently, cells were evaluated by immunofluorescence.

### Elyra 7 structured illumination microscope

A549 cells (50 × 10^3^ cells) were cultured in 24-well plates. Cells were imaged on the Elyra 7 Structured Illumination Microscope (Zeiss) in SIM mode, using a 100x oil-immersion objective and acquiring Z-stacks with a Z-step of 100 nm, covering the entire cell. The images were then processed using ZEN Black software (Zeiss) with SIM2 script (3D-leap mode, default settings for fixed samples; IκBα protein – weak signal settings, mitochondria – strong signal settings).

### Wound-healing assay

A549 and H460 cells infected with lentivirus carrying either IκBα-WT, IκBα-MTS, or a control were seeded in 6-well plates and cultured until reaching 90% confluence. Subsequently, the cells were starved for 24 h. After starvation, a wound was created in the center of the cell monolayer using a sterile plastic pipette tip. Serum-free medium was added to each well, and images of the wounds were captured at time 0 and after 15 h using live-cell imaging microscopy (Carl Zeiss). The percentage of wound closure was quantified using either the Axio Vision program or ImageJ with the Wound-Healing Size Tool.

### Anchorage-independent cell growth

Anchorage-independent cell-growth assays were conducted by suspending cells in 0.45% type VII low-melting agarose in 10% RPMI at a density of 5 × 10^3^ cells per well. These cell suspensions were then plated on a layer of 0.9% type VII low-melting agarose in 10% RPMI in 6-well plates and cultured at 37 °C with 5% CO_2_. After 2 weeks, colonies were counted, and images were captured at 5× magnification.

### Lactate measurement

The media were assayed for lactate levels using a Biosen C-Line analyzer according to the manufacturer’s instructions.

### Assessing cellular respiration in vitro

In vitro cellular respiration was measured by assessing the oxygen flux of proliferating A549 cells and those undergoing differentiation using a RESIPHER device from Lucid Scientific. A 32-sensor lid compatible with a 96-well plate was utilized for this purpose. IκBα-WT, IκBα-MTS, or control A549 cells were seeded at a density of 10^4^ cells per well. Oxygen consumption data were sampled every 3 min throughout the experiment.

### Statistical analysis

Data are presented as mean ± SEM. Statistical significance was determined using Student’s t-test or one-way or two-way analysis of variance (ANOVA), as appropriate, followed by Fisher’s least significant difference (LSD) or Bonferroni post hoc tests. The specific statistical tests used and the exact sample size (*n*) for each experiment are indicated in the corresponding figure legends. A *P* value < 0.05 was considered statistically significant (*P* ≤ 0.05; *P* ≤ 0.01; *P ≤ 0.001; **P ≤ 0.0001). All statistical analyses were performed using GraphPad Prism 9.

Further details on materials and methods can be found in the supplementary section

## Supplementary information

SUPPLEMENTAL MATERIAL

original data

## Data Availability

All data generated or analyzed during this study are included in this published article and its supplementary information and are available from the corresponding author on reasonable request.
