# Supplementary material for: Mitochondrial IκBα fuels cancer progression through metabolic rewiring, endothelial activation, and thrombotic spread
Source: Cell Death Discov. 2026 Mar 27;12:281. doi: 10.1038/s41420-026-03022-0 (PMC13287714; doi:10.1038/s41420-026-03022-0)
Supplement: Supplementary file 1 — SUPPLEMENTAL MATERIAL [file 41420_2026_3022_MOESM1_ESM.pdf]

SUPPLEMENTARY MATERIAL AND METHODS

Lentiviral transduction

Forty-eight hours after co-transfecting HEK293T cells with pCMV-VSV-G (Plasmid #8454), pCMV-dR8.2 dvpr (Plasmid #8455) and the shRNA vector, the viral supernatants were collected and used to infect the target cells. Following infection, the cells were selected using 1 μg/ml puromycin. IκBα-MTS was constructed using the Addgene vector #44652 as the template.

Antibodies

The following antibodies from cell signaling were used: IkappaB alpha (44D4) Rabbit Monoclonal Antibody #4812; IkappaB alpha (L35A5) Mouse Monoclonal Antibody (Amino-terminal Antigen) #4814; Phospho-IkappaB alpha (Ser32) (14D4) Rabbit Monoclonal Antibody #2859; NF-κB p65 (D14E12) XP® Rabbit mAb #8242; Resources results for "NF-κB p65 (D14E12) XP® Rabbit mAb; Phospho-IKKα/β (Ser176/180) (16A6) Rabbit mAb; VDAC (D73D12) Rabbit Monoclonal Antibody #4661. The following antibodies were used: vWF (Thermo Fisher, # MA5-14029); CD31 (abcam, ab28364). Vinculin Recombinant Rabbit Monoclonal Antibody (9I3Y8) - MA5-42795.

Tube formation

Matrigel Basement Membrane Matrix (BD Biosciences) was diluted with conditioned medium derived from cells expressing either IκBα-WT, IκBα-MTS, or a control, with or without silencing of p65, and coated onto 6-well plates. The plates were then incubated at 37°C for 1 hour. Subsequently, 5 × 10^4 HUVECs were seeded onto the coated plates. The tube formation ability of HUVECs was assessed after 6 hours of incubation, during which the total length of the tubular structures was quantified.

RNA extraction, cDNA synthesis, and qRT-PCR

RNA extraction from cells was performed using TRIzol, following the manufacturer's instructions. Then, 1 μg of total RNA was reverse transcribed into cDNA using the iScript cDNA Synthesis Kit (Bio-Rad), as per the provided protocol. Real-time PCR was conducted with TaqMan Real-Time PCR Assays under the following conditions: an initial denaturation step at 95°C for 3 minutes, followed by 40 cycles of 95°C for 15

seconds and 60°C for 30 seconds. The assays utilized in the experiment are as follows: VCAM1 / FAM-MGB (HS01003372_M1-4453320); ICAM1 / FAM-MGB (Hs00164932_m1-4453320); Vwf / FAM-MGB (Hs01109446_m1-4331182) (Life Technologies – Invitrogen)

Western Blot analysis, cell lysis and mitochondria isolation

Lung cancer cells were lysed in a buffer containing 150 mM NaCl, 1 mM EDTA, 50 mM Hepes (pH 7.5), 1% Triton X-100, and 10% glycerol, with added protease and phosphatase inhibitors. After lysis, the samples were spun down at 14,000 rpm for 15 minutes. The protein concentration was measured using the Bradford assay (Serva, # 39222.03). Between 30 and 50 µg of total protein from each sample were denatured in Laemmli buffer (2X) for 5 minutes, then analyzed via Western blotting using 4–15% polyacrylamide gels. After electrophoresis, proteins were transferred to 0.45 µm nitrocellulose membranes. The membranes were then subjected to immunoblotting, where they were incubated overnight at 4°C with specific primary antibodies (see below) in a solution of DPBS, supplemented with 0.1% Tween and 1% BSA. Detection of the target proteins was done using peroxidase-linked secondary antibodies and a chemiluminescent substrate (BIORAD, #170-5060). Mitochondrial extractions were carried out using the Mitochondria Isolation Kit for Cultured Cells (Thermo Fisher, # 89874), following the manufacturer’s protocol.

In vivo tumor and spontaneous metastasis

A total of 2.5 × 10^6 A549 cells infected with a lentivirus carrying either IκBα-WT, IκBα-MTS or with a control were subcutaneously inoculated into 6-week-old male nude mice(Male CR ATH HO MOUSE 42-48 Days). Upon tumor growth, reaching a volume of 25 mm^3, tumors were resected and left for 30 days. Subsequently, mice were sacrificed, and lung tissues were collected for further analysis. Lung sections were then stained with hematoxylin and eosin (H&E) and evaluated for metastases using a DM6 microscope (Leica Microsystems).

In vivo metastasis assays

The experimental metastasis assay involved the injection of 5 × 10^5 A549 cells infected with lentivirus carrying either IκBα-WT, IκBα-MTS, or a control into the tail vein of 6-week-old male immunodeficient NSG mice (Male NSG MOUSE 42-48 Days). After

24 days, the animals were sacrificed, and their lungs were collected, formalin-fixed, and paraffin-embedded (FFPE). Lung sections were then stained with hematoxylin and eosin (H&E) and evaluated for metastases using an Olympus BH2 microscope across the entire lung section by ImageJ Software (Universal Imaging Corporation).

In vivo administration of cancer cell conditioned medium

A549 cancer cells (empty vector, IκBα-WT, and IκBα-MTS) were initially seeded in 10-cm petri dishes and allowed to grow for 24 to 72 hours. Following this, the medium was replaced with RPMI devoid of glutamine and phenol red (Gibco, 32404014), and cells were then incubated for an additional 48 hours. Subsequently, conditioned medium was collected and centrifuged at 3130xg for 10 minutes. The resulting supernatants were supplemented with Hepes (20 mM) and filtered using a 0.22 μm filter. Next, 7-week-old nude mice (Male CR ATH HO MOUSE 42-48 Days) were intravenously injected with 300 μl of conditioned medium every 2 days for a duration of 24 days. Following the preconditioning with the conditioned medium, A549 cells were injected into the mice via the tail vein. Mice were euthanized 30 days after the initial injection, and their lungs were collected and subjected to formalin fixation and paraffin embedding (FFPE). Lung sections were subsequently stained with hematoxylin and eosin (H&E) and assessed for metastases using an Olympus BH2 microscope. Image analysis was performed using ImageJ Software (Universal Imaging Corporation) across the entire lung section.

Sample size per group and randomization

Animals were used in accordance with Animal Welfare Protocol No. 219/2023-PR (ref. CC652.196). Sample size was calculated to detect a 30% difference in metastatic burden between experimental groups, assuming a standard deviation of 20%, with a two-sided significance level of 0.05 and 80% power. Based on these parameters, 6 mice per group were required.

Animals were randomly assigned to experimental groups to ensure that the groups were balanced for weight, age, and overall health status.

No blinding was performed; group allocation was known during both the experiments and outcome assessment

Cancer cell adhesion assay

HUVECs were seeded in 96-well plates as previously described in the in vitro gene silencing procedure, and the adhesion assay was performed on day 6. A549 cells, infected with lentivirus carrying either IκBα-WT, IκBα-MTS, or a control and expressing GFP were harvested, counted, and resuspended in M199 medium (Gibco, 12340-030) supplemented with 1% BSA. After completely removing the supernatant from HUVECs, cancer cells were added on top (40,000 cells per well) and allowed to adhere for 1 hour at 37°C with 5% CO2. Subsequently, non-adherent cells were eliminated by washing twice with PBS. For microscopic evaluation, adherent cancer cells were fixed and stained with DAPI. For FACS analysis, HUVECs were collected with trypsin, transferred to a V-bottom 96-well plate, centrifuged at 360g for 5 minutes at 4°C, and fixed in a 1:1 mixture of IC fixation buffer (eBioscience, 00-8222-49) and FACS buffer. Precision count beads (BioLegend, 424902) were added before analysis of tdTomato+ cells using a Fortessa LSR-II (BD Biosciences).

Immunofluorescent staining for vWF/CD31

Tissues embedded in OCT were sliced into 7 μm thick sections using cryo-sectioning. The resulting slides were air-dried completely before fixation with 4% PFA for 10 minutes at room temperature. Subsequently, the slides were rinsed three times in phosphate-buffered saline (PBS) containing 0.1% Triton X-100 (PBST) at neutral pH for 5 minutes each. The sections were then surrounded by PAP-PEN, and a blocking buffer (10% horse serum in PBS) was added to them for 1 hour at room temperature in a humidified chamber. After draining off the blocking buffer, primary antibodies at 1/100 dilution in PBS and CD31 at 1/250 dilution in PBS) were added and left overnight at 4°C. The next day, the slides were washed three times for 5 minutes each using PBS containing 0.05% Tween-20. Subsequently, the secondary antibody (Anti-rat Alexa-fluor 488) diluted at 1/500 in PBS was added for 60 minutes at room temperature. Following another three washes of 5 minutes each using PBS containing 0.05% Tween-20, DAPI (1:1000 dilution) was added for 15 minutes, followed by repeated washes. Finally, the slides were mounted with ProLong Gold Antifade Mountant for further analysis.

Immunofluorescence

A549 cells (50 × 10^3) infected with lentiviruses expressing either IκBα-WT, IκBα-MTS, or a control were cultured in 24-well plates. After fixation with 4% paraformaldehyde (PFA) and blocking with 1% BSA in PBS, cells were stained for recombinant IκBα

proteins localized to the mitochondria using a primary antibody, which was incubated for 2 hours at room temperature. Following this, Mitotracker was added according to the manufacturer's instructions, and DAPI was used for nuclear staining. The samples were imaged using Z-stack confocal microscopy (Leica SP8).

For immunofluorescence assays, human umbilical vein endothelial cells (HUVECs) were seeded into 24-well plates at a density of 50 × 10^3 cells per well. Conditioned medium from A549 cells infected with lentiviruses carrying IκBα-WT, IκBα-MTS, or a control was then applied to the HUVECs. After 24 hours of treatment, the cells were fixed with 4% PFA and blocked with 1% BSA in PBS. Cells were then stained for vWF using a primary antibody, incubated for 2 hours at room temperature. After applying a secondary antibody and performing DAPI staining, the samples were analyzed using Z-stack confocal microscopy (Leica SP8).

Transwell Assay and Endothelial Cells Transwell Assay

A549 and H460 cells infected with lentiviruses carrying either IκBα-WT, IκBα-MTS, or a control were first seeded into 6-well plates. After a 24-hour starvation period, the starved cells were transferred to the upper chambers of Transwell inserts, with the lower chambers filled with medium. After incubating for 12 hours to allow migration, cells on the membrane were fixed and stained. The number of migrated cells was determined either by measuring absorbance at 500 nm or by visual counting under a microscope. Images were captured using an Olympus BH2 microscope, and analysis was conducted with ImageJ software (Universal Imaging Corporation).

For the endothelial cell Transwell assay, HUVECs were seeded into the upper chambers, while A549 cells (infected with lentiviruses carrying either IκBα-WT, IκBα-MTS, or a control and expressing GFP) were cultured in 6-well plates and subjected to a 24-hour starvation period. Following starvation, these cells were transferred to the upper chambers of Transwell inserts placed in wells containing medium in the lower chamber. After 12 hours of incubation, the cells were fixed, stained, and the number of migrated cells was counted under a microscope.

SUPPLEMENTARY FIGURES

SUPPLEMENTARY FIGURE 1

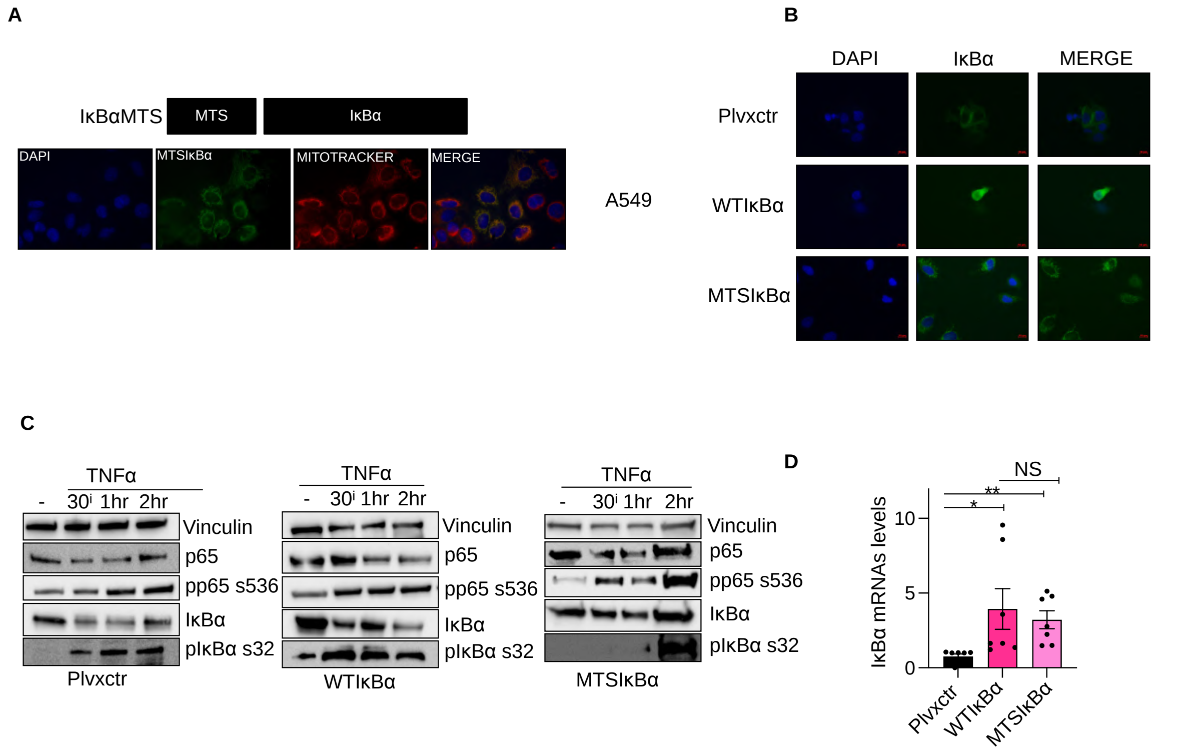

Supplementary Figure 1: Stabilization of IκBα in the mitochondria

(A) Immunofluorescence analysis of A549 cells infected with MTS-IκBα (green), co-stained with MitoTracker (red).(B) Confocal immunofluorescence analysis of A549 cells infected with control, WT, or MTS-IκBα (green), demonstrating distinct vector localization.(C) Western blot analysis of A549 cells infected with control, WT, or MTS-IκBα, treated with TNFα for the indicated durations.(D) Quantitative RT-PCR analysis performed on A549 cells which were infected with control vector, WT, or MTS IκBα. Graph shows means ± SEM; (n= 7), P-values are from Student's t-test. *P < 0.05; **P < 0.01; WT and MTS indicate overexpression constructs for wild-type IκBα and mitochondrially targeted IκBα, respectively. "Control" refers to cells expressing endogenous IκBα only.

SUPPLEMENTARY FIGURE 2

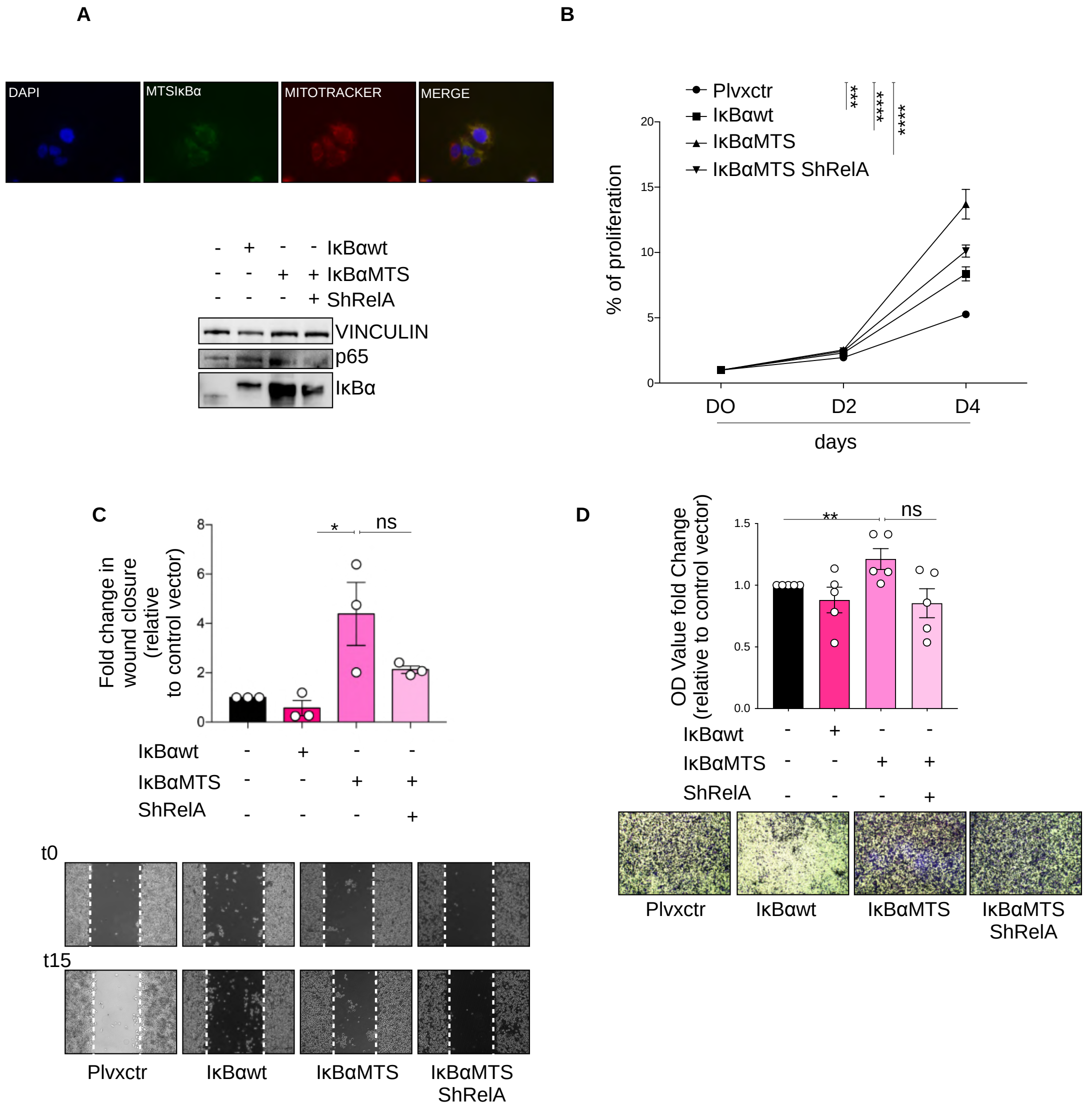

Supplementary Figure 2: Augmented mitochondrial IκBα expression boosts the aggressiveness of lung cancer cells

(A) Upper pnanel: Immunofluorescence analysis of A549 cells infected with MTS-IκBα (green), co-stained with MitoTracker (red). Lower panel: Western blot analysis of IκBα and p65 in H460 cells demonstrating the overexpression of wt and MTS variants compared to control cells, along with p65 silencing. (B) Proliferation was evaluated over 4 days using the Cell Titer Glo assay. Graph shows means ± S.E.M.; N=3, P-values are from Student's t-test. ***$P < 0.001$; ****$P < 0.0001$. (C) Upper Panel: Fold change of wound closure, illustrating the progression of wound healing at 0 hours and 15 hours post-wounding. Lower Panel: Representative pictures captured at the same

time points to offer visual insight into the dynamic healing. (D) Invasion assay, shown as fold change relative to control cells. Representative images of cells that have invaded through the membrane, stained with crystal violet are shown. Graph shows means ± S.E.M, N=4. WT and MTS indicate overexpression constructs for wild-type IκBα and mitochondrially targeted IκBα, respectively. “Control” refers to cells expressing endogenous IκBα only.

SUPPLEMENTARY FIGURE 3

A

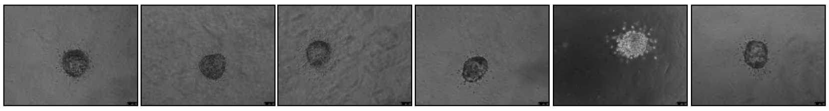

B

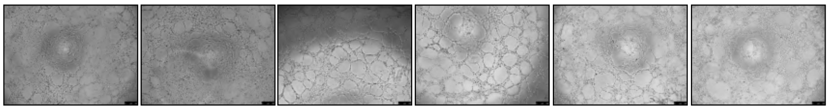

Supplementary Figure 3: Effect of IκBα Overexpression on angiogenesis

(A) Representative images of the sprouting formation assay. Endothelial cells were embedded in a three-dimensional matrix and incubated for 6 hours with conditioned medium (CM) derived from A549 cells infected with control vector, WT, or MTS IκBα. Sprout formation was visualized by phase-contrast microscopy. Quantification of sprout length and number of branch points was performed using image analysis software. **(B)** Representative images of tube formation assay. Endothelial cells were plated on Matrigel and incubated for 6 hours in conditioned medium (CM) derived from A549 cells infected with control vector, WT, or MTS IκBα. The formation of capillary-like structures was visualized using phase-contrast microscopy. Quantification of tube length and branch points was performed using image analysis software.
