# Supplementary figures and images for: Mitochondrial IκBα fuels cancer progression through metabolic rewiring, endothelial activation, and thrombotic spread
Source: Cell Death Discov. 2026 Mar 27;12:281. doi: 10.1038/s41420-026-03022-0 (PMC13287714; doi:10.1038/s41420-026-03022-0)

FIGURE 1A

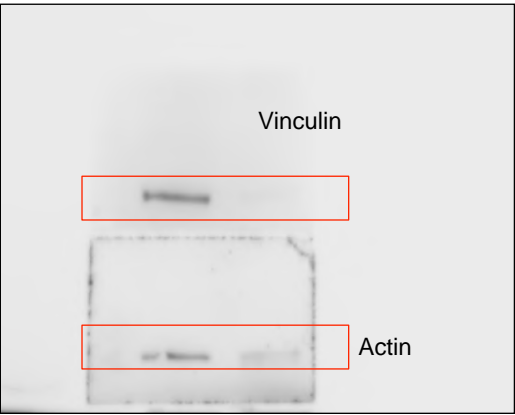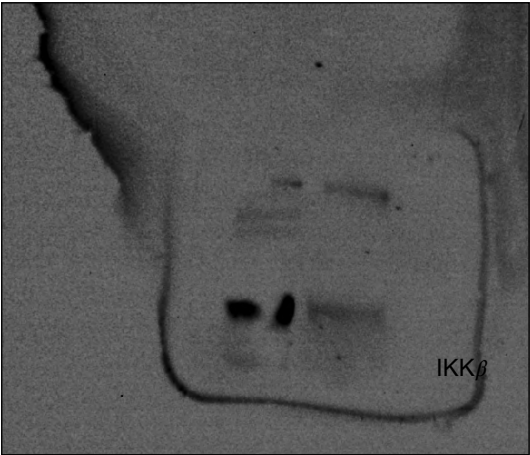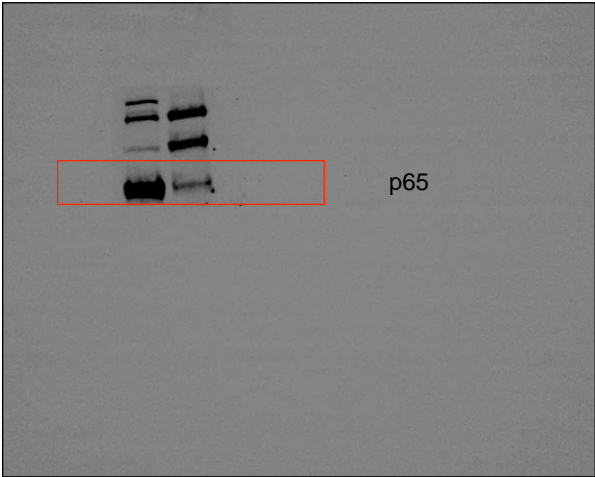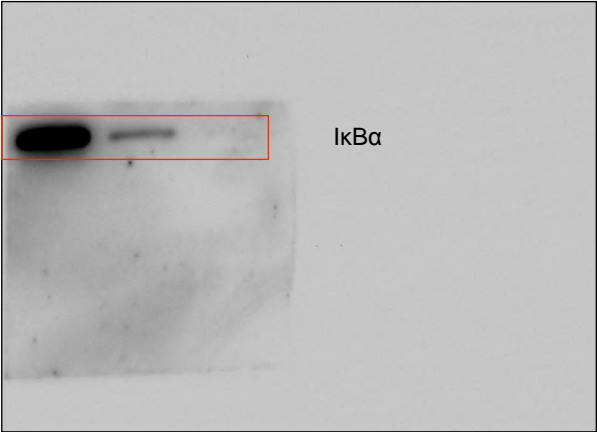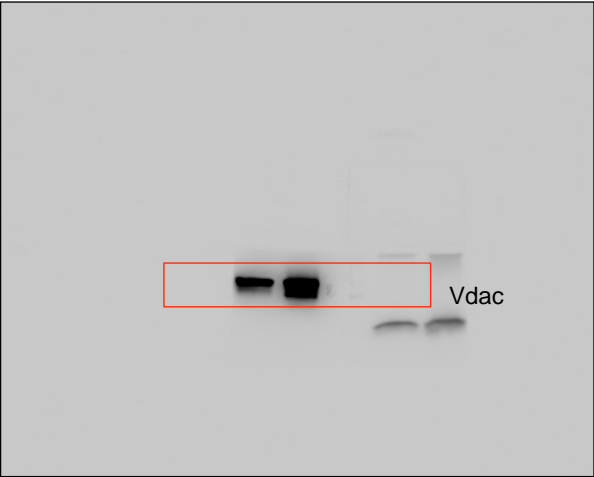

FIGURE 2A

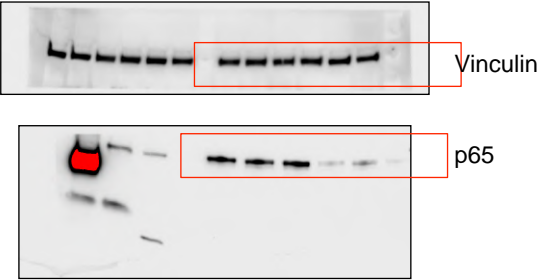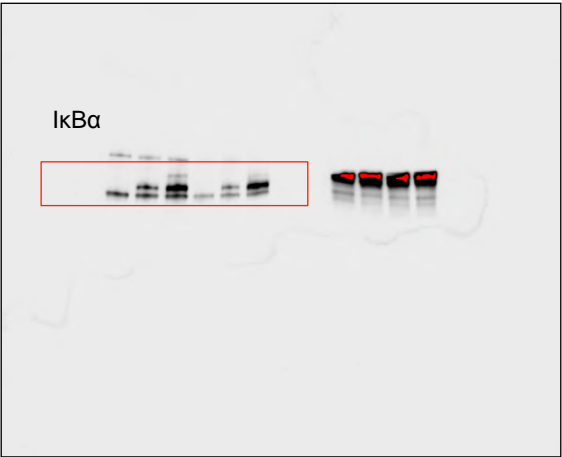

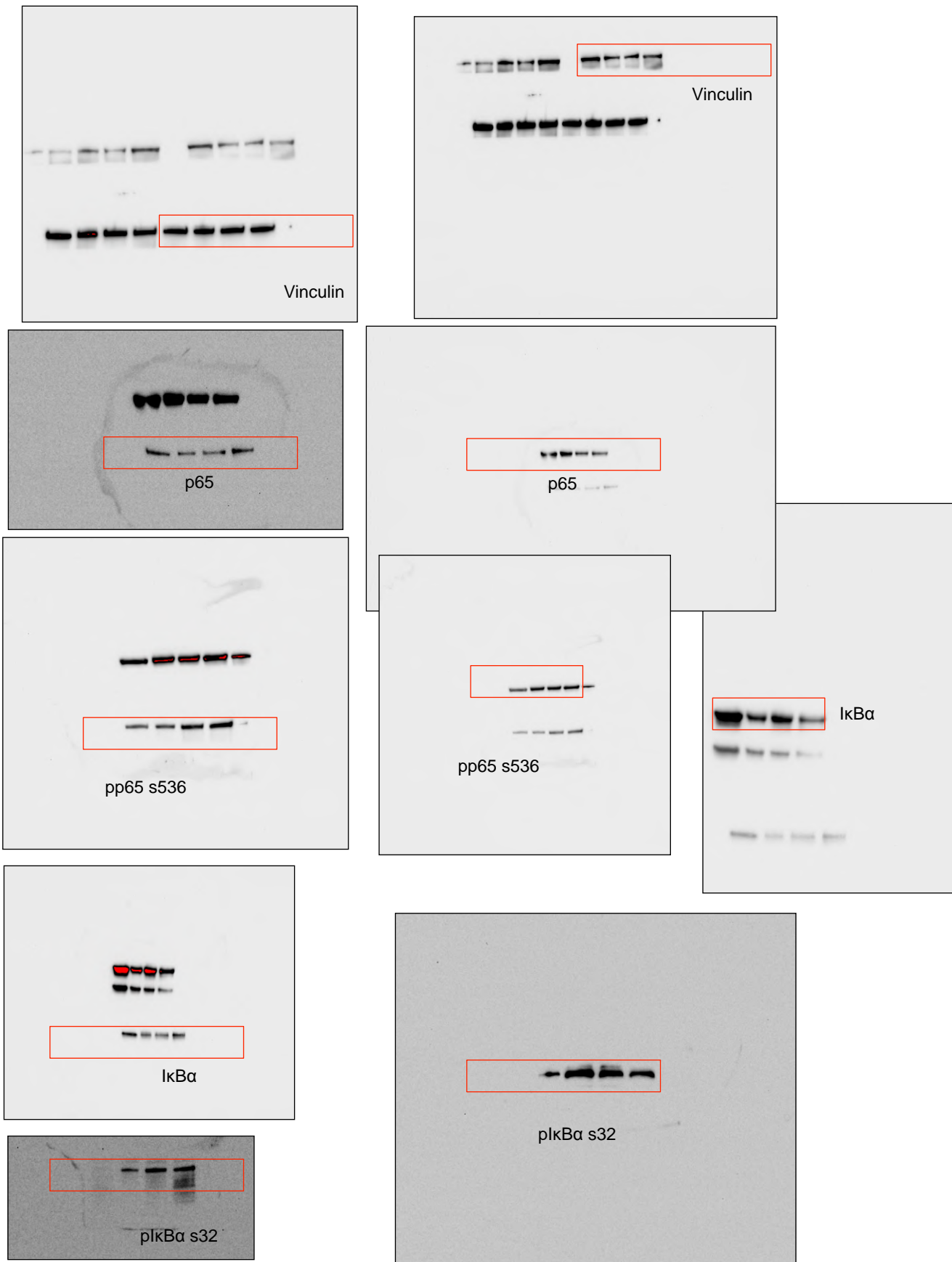

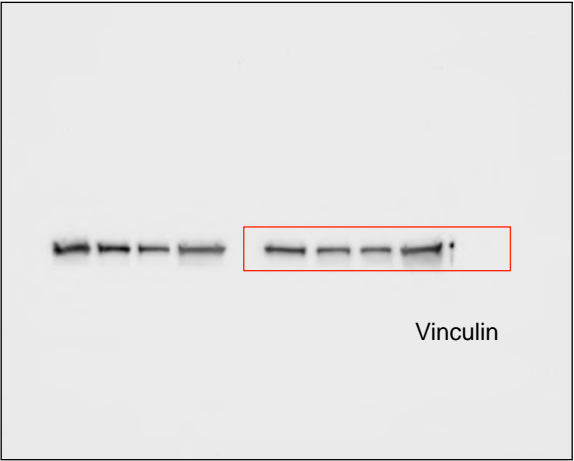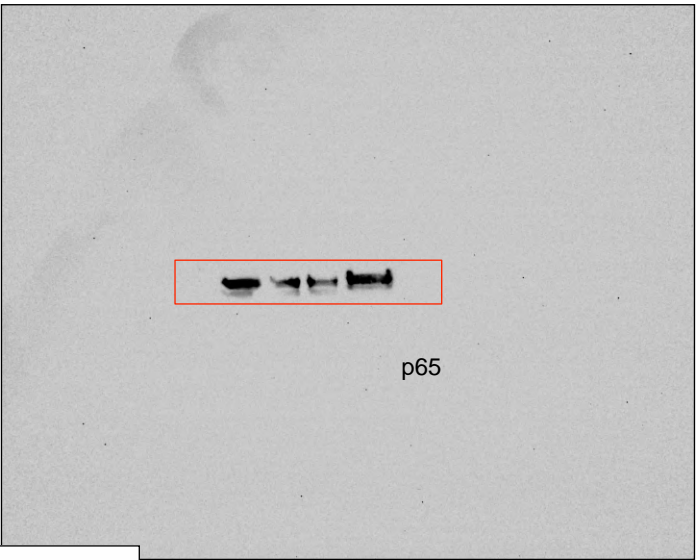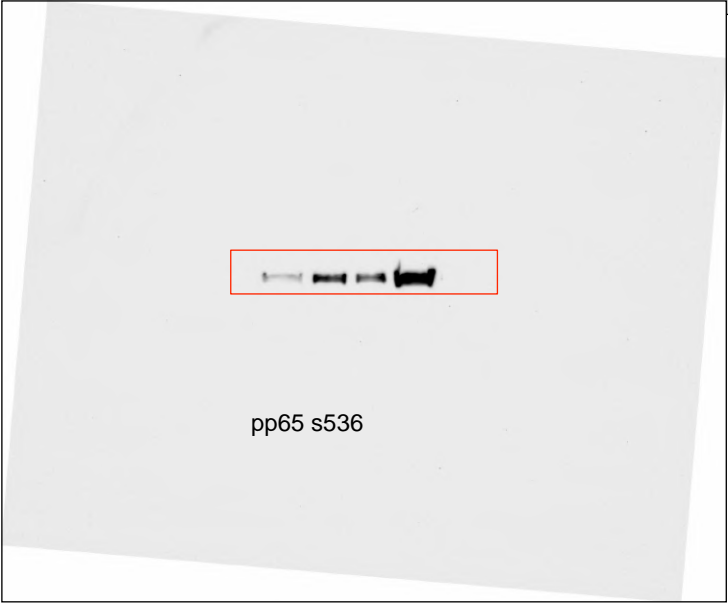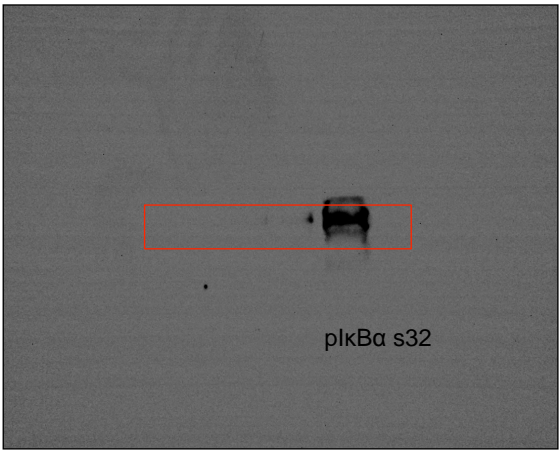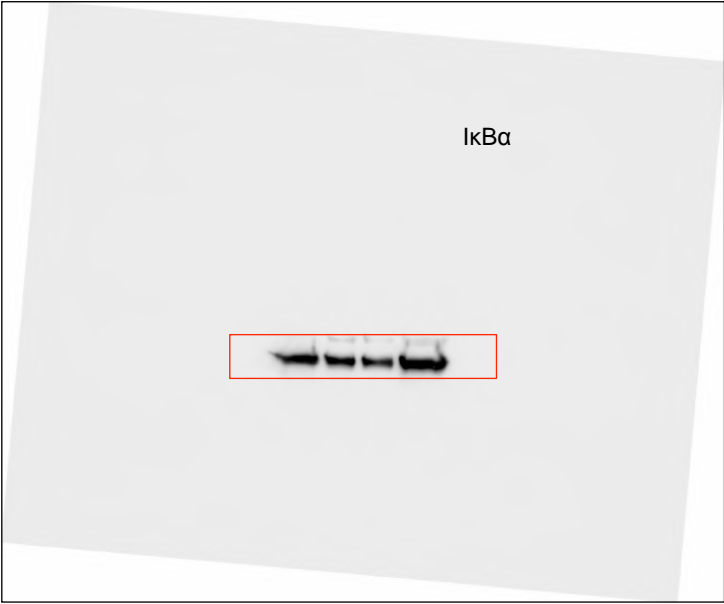

Supplement: Supplementary file 2 — original data [file 41420_2026_3022_MOESM2_ESM.pdf]
